# Quality of life following treatment with intra-arterial cisplatin with concurrent radiation and erlotinib for locally advanced head and neck cancer

**DOI:** 10.1007/s00520-023-08286-1

**Published:** 2024-01-09

**Authors:** Ricardo Cossyleon, Kathy Robinson, Kristin Delfino, K. Thomas Robbins, Krishna Rao

**Affiliations:** 1 Simmons Cancer Institute, Southern Illinois University School of Medicine, 315 W. Carpenter St., PO Box 19677, Springfield, IL USA; 2 Center for Clinical Research, Southern Illinois University School of Medicine, Springfield, IL USA; 3 Division of Hematology and Medical Oncology, Department of Internal Medicine, Southern Illinois University School of Medicine, Springfield, IL USA; 4 Division of Otolaryngology Head and Neck Surgery, Department of Surgery, Southern Illinois University School of Medicine, Springfield, IL USA; 5 Department of Medical Microbiology, Southern Illinois University School of Medicine, Springfield, IL USA

**Keywords:** Head and neck cancer, Quality of life, Intra-arterial cisplatin, Erlotinib, Chemoradiation

## Abstract

**Objectives:**

Studies that focus on the feasibility of using erlotinib plus chemoradiation to treat locally advanced head and neck cancer have given hints of improved survival outcomes compared to chemoradiation alone. However, the influence of this treatment regimen on the quality of life of the patients has not been documented. We conducted a study of this triple combination and now have documented follow-up survival data as well as long-term quality of life (QoL) measures.

**Methods:**

Three sets of QoL questionnaires were given to patients with a diagnosis of head and neck cancer at two time points, pre- and post-treatment, to assess differences in quality of life after receiving chemotherapy with intra-arterial (IA) cisplatin (150 mg/m^2^), concomitant radiation (70 Gy), and oral erlotinib (150 mg/day). Additionally, patients were followed for a total of 5 years.

**Results:**

Treatment had a detrimental effect on appearance, taste, and saliva domain scores in their QoL questionnaires. Nonetheless, fewer patients reported pain and anxiety.

**Significance of results:**

The combination of erlotinib with chemoradiation produced similar adverse effects on the QoL scores of patients with head and neck cancer as compared to chemoradiation alone.

**Supplementary Information:**

The online version contains supplementary material available at 10.1007/s00520-023-08286-1.

## Introduction

Although treatment advances for head and neck cancer (HNC) have increased survival rates, these advances may have a detrimental effect on other domains [1]. For instance, it is of preference for physicians to opt for organ preservation treatments (non-surgical interventions); however, these may increase the risk of having functional disorders, such as swallowing difficulty due to dysfunction of oral structures [2]. Therefore, clinical improvement does not necessarily correlate with better quality of life (QoL). For this reason, the use of different QoL questionnaires in patients with cancer allows us to measure the subjective impact of the disease on different domains of the patient’s daily routine, including their emotion towards their current diagnosis [3]. Additionally, when two or more treatment options offer similar survival rates, one may use QoL assessments to select which treatment would offer the most benefit overall [1].

Many studies have shown that the addition of several non-invasive treatments may have a synergic effect in the overall survival of patients [4–6]. However, new studies have focused on modifying treatment regimens to not only increase survival, but to also reduce toxic effects [7]. Therefore, there is a fine line to decide if another combination of agents should be added or if the current regimen should be modified instead.

Since over 90% of the HNC malignancies express an epidermal growth factor receptor (EGFR), there is an increased focus to include biological agents that block this receptor and to compare their outcome to the standard of care [6, 8–10]. Recently, along with other studies, we demonstrated the feasibility of combining an EGFR receptor therapy agent, erlotinib specifically, with chemoradiation [6]. This combination presented hints of improved survival outcomes 2 years post-treatment. However, we are now left with the question of whether this treatment regimen may or may not affect the QoL of the patients along a 5-year follow-up.

## Methods

The data used for this study was obtained from 21 patients who were enrolled between May 2006 and May 2010 to an open-label, non-randomized, phase II study by Dr Rao et al. [6]. Subjects eligible for this study included patients with untreated, biopsy-proven, stage III or IV, T3–T4 squamous cell carcinoma of the head and neck (oropharynx, hypopharynx, or larynx) with N0–N2 locally advanced diseases. Moreover, patients with unresectable tumors, such as T4b or N3 neck disease, were excluded from the study. This project was approved by the Springfield Committee for Research Involving Human Subjects (SCHRIS), submitted to the FDA under IND number 74,135, and registered at clinicaltrials.gov with number NCT00304278. Additionally, we obtained approval by the SIU Medicine IRB (IRB protocol 18–312) to perform a retrospective analysis of the survival/relapse data available from these patients to complete a 5-year follow-up. We conducted all procedures following the regulations established by SCRIHS and the Code of Federal Regulations, 20CFR Part 46.

Patients enrolled in the study had to complete the following three QoL questionnaires during pre- (week 0) and post- treatment (week 13) clinical visits: the Functional Assessment of Cancer Therapy Quality of Life Measurement System (FACT-H&N v4), the University of Washington Quality of Life Questionnaire (UW-QOL v4), and the MD Anderson Dysphagia Inventory. These questionnaires helped us to evaluate the patients’ health-related QoL, such as social interactions, pain, swallowing, mood, and anxiety. In detail, the FACT-H&N contains 38 items related to physical (7), social/family (7), emotional (6), and functional (7) well-being, plus “additional concerns” (11), such as “I am able to eat the foods that I like,” “My mouth is dry,” “I have trouble breathing,” “My voice has its usual quality and strength,” “I am able to eat as much food as I want,” “I am unhappy with how my face and neck look,” “I can swallow naturally and easily,” “I am able to communicate with others,” and “I can eat solid foods.” For each question, there are five possible answers that range from “not at all” (score 0) to “very much” (score 4). The score of each domain is then summed to display a composite QoL score. Higher scores represent better QoL [11]. The UW-QOL contains 12 domain-specific questions that allow the patients to describe their current pain, appearance, activity, recreation, swallowing, chewing, speech, shoulder limitations, taste, saliva, mood, and anxiety. Answers range from “very limited/affected by disease” (score 0) to “not limited at all/same as always” (score 100). As with FACT-H&N, the domain-specific scores are then summed into a composite score in which higher scores represent better QoL. Additionally, the UW-QOL includes a multiple choice question through which the patients may select up to three domains that they believe have been the most affected during the past week and three questions to compare their current health-related QoL with the previous week and month [12]. Lastly, the MD Anderson Dysphagia Inventory assesses if, and how severely, their dysphagia may affect their day-to-day activities. This questionnaire contains 20 questions that measure physical (8), emotional (6), functional (5), and global (1) swallowing-related QoL. Each question has five possible answers that range from “strongly agree” (score 1) to “strongly disagree” (score 5) [13]. The global answer is scored individually,the composite score is calculated with the mean of the other domains, multiplied by 20. Higher scores represent higher functioning QoL. To compare the score differences between pre- and post-treatment questionnaires, we analyzed the data with either paired *t*-test or Wilcox signed-rank test if data violated Shapiro–Wilk normality assumption.

All patients were set to receive treatment with 4 weekly infusions of intra-arterial cisplatin (150 mg/m^2^), intravenous sodium thiosulfate as neutralization of systemic cisplatin, and concomitant radiation (total dose of 70 Gy). Additionally, patients took erlotinib 150 mg q.d. during the 7 weeks of radiation. Radiation was given at 2 Gy/fraction per day, 5 days a week, to a total dose of 70 Gy through 7 weeks. Moreover, intermediate-risk regions received 60 Gy/35 fractions, while elective region received 54–56 Gy/35 fractions. Additionally, intensity modulated radiation therapy (IMRT) was utilized provided subject met necessary 3D-conformed treatment objectives. Complete treatment protocol and tumor response assessment can be found described in detail elsewhere [6]. Patients were assessed weekly using the ECOG performance status until week 7 of treatment initiation,assessments were then performed every 2 weeks until week 13. We used the Kaplan–Meier estimate analysis to calculate the overall survival and disease-free probabilities [14].

## Results

A total of 21 patients provided their written informed consent; 19 of these patients received at least 1 cycle of chemotherapy and were considered evaluable. Of the two patients who did not receive therapy, one could not obtain insurance approval, and the other was found to have a blockage of the common iliac arteries. After initiating treatment, two other patients were withdrawn from the study; one due to non-compliance; and the other due to the inability to tolerate the treatment. Moreover, after completing treatment, only 14 patients returned their post-treatment QoL questionnaires. Unfortunately, due to the fact that HPV testing was not performed regularly at the time, only two patients were tested and were HPV-negative.

The mean age at enrollment of the 19 evaluable patients was 50.8 years (range 33–67 years) with 89.5% of them being male. Additionally, 21% of the patients were African Americans, while the rest were Caucasians. Furthermore, 57.9% of the patients had a tumor stage of T4 at diagnosis, and 42% of the tumors were moderately differentiated (see Table 1 and 2). The average size of the primary tumor at diagnosis was 3.35 cm (range 1–6.37 cm) with 63% arising from the oropharynx, 21% from the supraglottic larynx, and 16% from the hypopharynx.

**Table 1:** TNM staging at enrollment. The number of patients is inside the parentheses

TNM stage									
	N0		N1		N2				
					a	b		c	
T3	5.3%	(1)	15.8%	(3)	-	15.8%	(3)	5.3%	(1)
T4	5.3%	(1)	10.5%	(2)	-	31.6%	(6)	10.5%	(2)

**Table 2:** Tumor grade at enrollment. The number of patients is inside the parentheses

Tumor grade			
Well, moderate	5%		(1)
Moderate	42%		(8)
Moderate, poorly	32%		(6)
Poorly	21%		(4)
			*(n)*

**Fig. 1:**
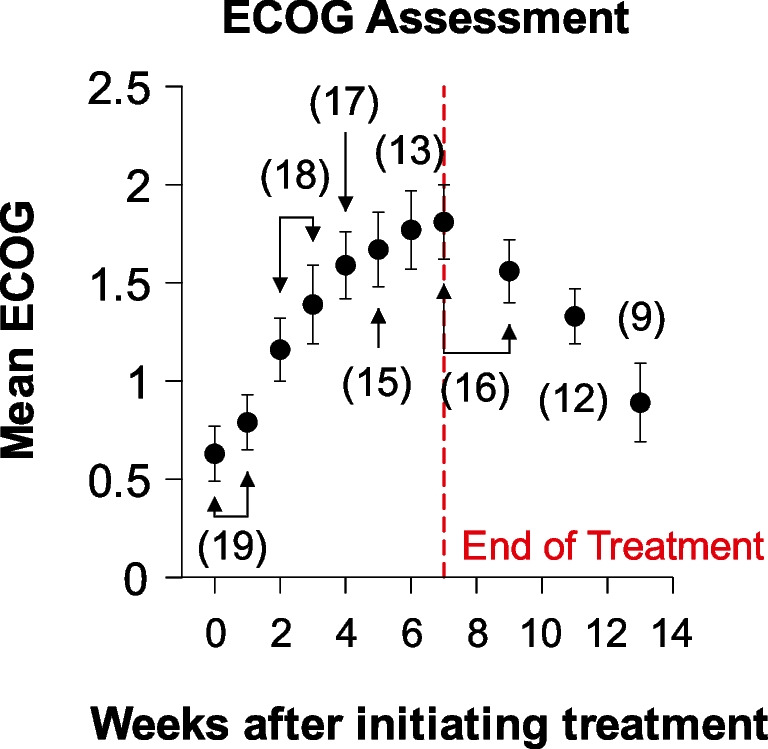
Mean ECOG performance status during treatment. The number of patients is inside the parentheses

During treatment, 18 patients were irradiated definitively with 12 of them receiving IMRT (6 of the patients through static fields, while the other 6 with a volumetric arc technique). The other six patients were treated using a custom blocking/compensation technique. The ECOG performance status of the patients increased from an average of 0.63 to a peak of 1.81 at week 7 (last week of treatment). Afterwards, the scores slowly decreased back to baseline at week 13 (Fig. 1). Moreover, 14 patients required the use of a gastrostomy feeding tube for an average time of 26.5 weeks. Two of these patients required prolonged tube feedings that lasted 79 and 91 weeks due to nausea and aspiration pneumonia with bronchopleural fistula, respectively. Additionally, another two patients had to have their feeding tube reinserted; one patient required feeding tube reinsertions during week 57–78 and 132–208 due to a dysfunctional larynx with a neck abscess draining through a pharyngocutaneous fistula; and the other patient at week 53 secondary to malignancy recurrence.

**Fig. 2:**
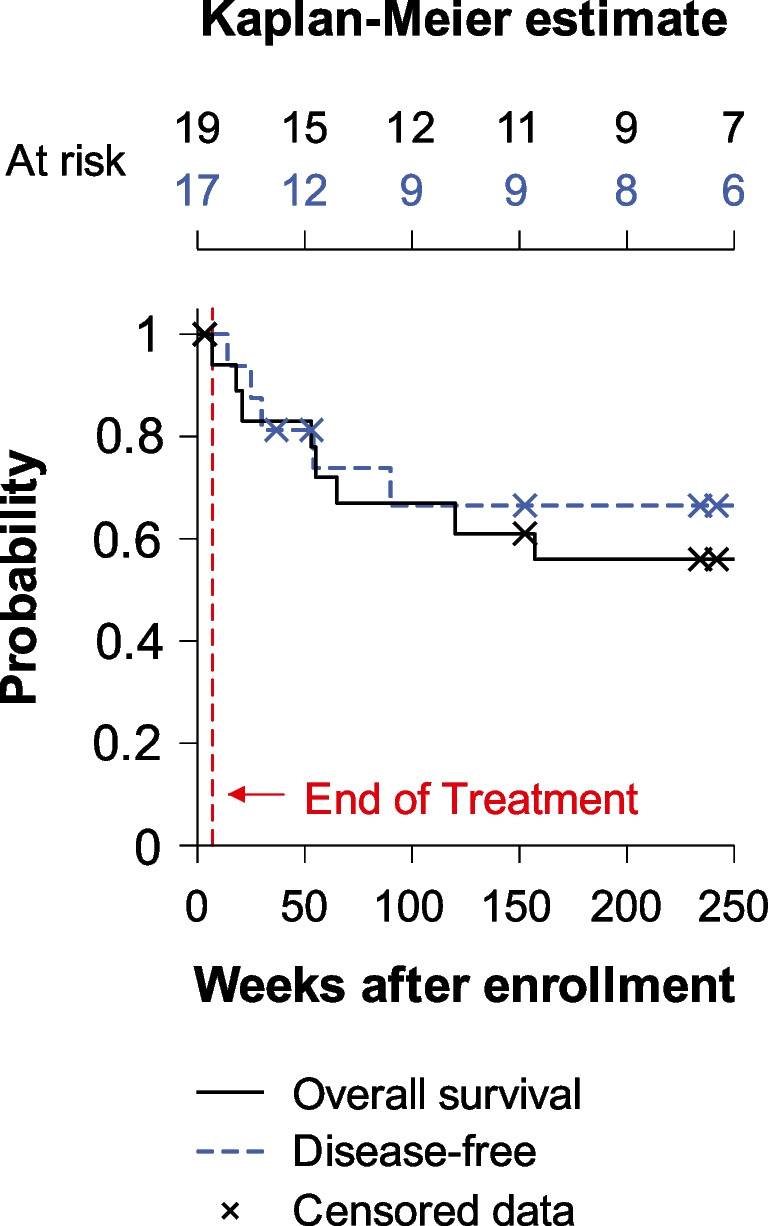
Overall survival (black continuous line) and disease-free (blue dashed line) survival probabilities. Data was estimated using the Kaplan–Meier survival formula

**Fig. 3:**
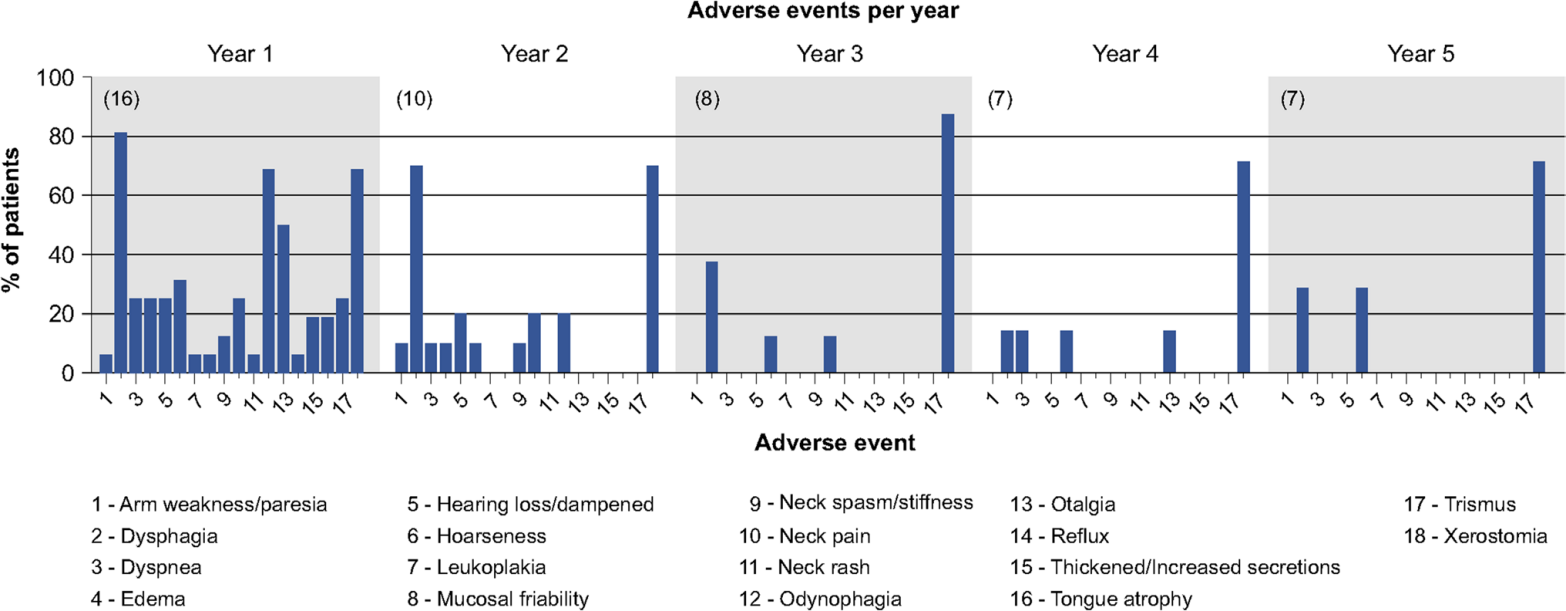
Common signs/symptoms observed in our patients following treatment. No bar symbolizes that none of the surviving patients reported that particular sign/symptom. The number of patients is inside the parentheses

After receiving treatment with IA cisplatin, IV sodium thiosulfate, and concomitant radiation plus erlotinib, 17 patients were restaged and followed for up to 5 years. During this period, 64.71% of the patients had complete remission and remained disease-free, while 11.76% had disease persistence. The remaining 23.53% of the patients had one or multiple recurrences and required more invasive interventions. One patient passed away at week 53 without evidence of local or distant recurrence. To calculate the overall survival of the 19 evaluable patients, we used the time of enrollment until death or last known follow-up, with the latter being censored (Fig. 2). Our population had a median survival time of 398 weeks (7.6 years) and a 5-year survival rate of 55% (Std Err = 11.86%) with 9 censored patients (median follow-up time of 265 weeks). Likewise, disease-free survival was calculated from the time of enrollment until evidence of recurrence or last known follow-up (censored). Two of the 19 evaluable patients were not included in this sub-analysis due to persistent disease. Moreover, median disease-free survival time could not be determined as 50% of the patients did not have disease recurrence. The 5-year disease-free survival rate of our patients was 66.48% (Std Err 12.37%) with 12 censored patients (median follow-up time of 253 weeks). Patients with recurrent/persistent disease only presented local lesions without evidence of any distant metastasis. Next, we collected any documented sign/symptom that was associated with their treated malignancy during these 5 years or until they presented with evidence of disease recurrence. This data showed that over 50% of the patients complained of dysphagia, odynophagia, otalgia, and xerostomia during the first-year post-treatment and improved with time. However, over 68% of the patients continued to report xerostomia for at least 5 years post-treatment (Fig. 3). If we group the patients by the type of radiation they received, we noticed that 83.3% of the patients who underwent radiotherapy with custom blocking reported xerostomia in the first-year post-treatment compared to only 41.6% of the patients who underwent IMRT. Additionally, 66.6% of the patients who underwent custom blocking radiotherapy did not survive more than 2 years post-treatment. Moreover, of all the patients, three had a chronic oral lesion with bone exposure after completing treatment, and one reported loss of taste. Additionally, three patients required multiple esophageal dilatations for strictures, and one had a recurrent pharyngocutaneous fistula and a recurrent neck abscess.

**Fig. 4:**
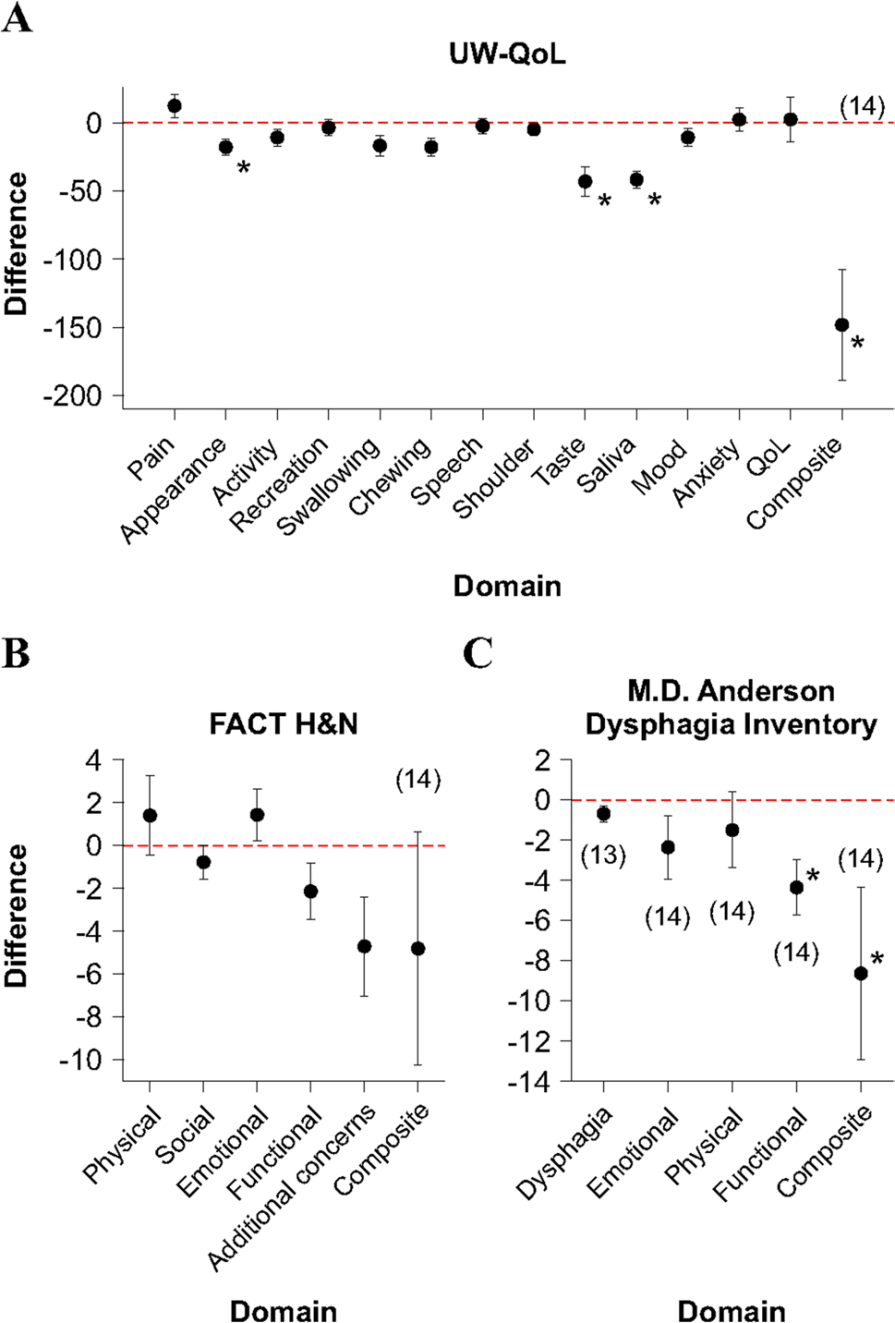
Difference between pre- and post-treatment scores using the **A** UW-QoL, **B** FACT-H&N, and **C** MD Anderson Dysphagia Inventory questionnaires. Positive values represent improvement post-treatment. The number of patients is inside the parentheses

The use of QoL questionnaires allowed us to evaluate the status of patients through different functional domains. Since we had the patients complete a questionnaire pre- and post-treatment, we were able to use their pre-treatment scores as their own control to measure any difference that the treatment had on their QoL. The average composite UW-QOL score from the 14 patients that completed both sets of questionnaires was 850.57 (range 659–1142) pre-treatment and 702.29 (range 458–834) post-treatment (for individual results, see supplementary Table S1). This represents a score decrease of 148.29 points (paired *t*-test, *P* < 0.01). Additionally, if we separate the score for each domain, we saw that the domains for pain and anxiety had a score increase of 12.5 (*P* = 0.16) and 2.43 (*P* = 0.77), respectively. However, there was a decrease in the score of all other domains (appearance, 17.86, paired *t*-test *P* = 0.01; activity, 10.71, Wilcox signed-rank *P* = 0.18; recreation, 3.57, *P* = 0.76; swallowing, 16.71, *P* = 0.10; chewing, 17.86, *P* = 0.06; speech, 2.29, *P* = 1.0; shoulder, 4.79, *P* = 1.0; taste, 43, *P* < 0.01; saliva, 41.67, *P* < 0.01; and mood, 10.71, *P* = 0.21; Fig. 4A). In agreement with this, in the section where the patients may specify up to three domains that they consider the most affected by their disease, more people selected swallowing, chewing, taste, saliva, and mood on their post-treatment questionnaire compared to their response pre-treatment (see Table 3). Furthermore, when asked if the patients believed that their overall health-related QoL changed during the past month, their average score increased by just 2.5 points (paired* t*-test, *P* = 0.88).

**Table 3:** Major complaint while answering the UW-QoL questionnaire. This represents the sum of patients that selected a particular symptom in a multiple choice question. Patients had to select up to three symptoms. The number of patients is inside the parentheses

	Pre-treatment	Post-treatment
Pain	9	3
Appearance	0	4
Activity	4	3
Recreation	0	0
Swallowing	9	13
Chewing	3	7
Speech	4	1
Shoulder	0	0
Taste	2	6
Saliva	3	4
Mood	0	1
Anxiety	5	1
		(14)

Another widely used questionnaire to assess the QoL of patients with HNC malignancies is the FACT-H&N QoL. The average composite score from this questionnaire was 93.91 (range 65–134) pre-treatment and 89.09 (range 49–119) post-treatment (for individual results, see supplementary Table S2). This represents a decrease of 4.81 points (paired *t*-test, *P* = 0.39) following treatment. However, if we separate the score for each domain, we observed that the scores for the physical and emotional domains increased by 1.40 (*P* = 0.46) and 1.43 (*P* = 0.25), respectively (Fig. 4B), while the social/family, functional, and “additional concerns” decreased by 0.78 (*P* = 0.33), 2.14 (*P* = 0.12), and 4.71 (*P* = 0.06).

Lastly, we provided the MD Anderson Dysphagia Inventory for the patient to answer pre- and post- treatment. With this instrument, we observed that the average global dysphagia assessment pre-treatment was 3.15 and 2.46 post-treatment (for individual results, see supplementary Table S3). This yields a decrease of 0.69 points (Wilcox signed-rank, *P* = 0.11). Moreover, when analyzing the composite assessment, there was a decrease of 8.64 points (Wilcox signed-rank, *P* = 0.01). As with the other questionnaires, we separated the score for each of the domains, and we saw that there was a decrease in the emotional (2.36, paired *t*-test, *P* = 0.16), physical (1.50, *P* = 0.44), and functional (4.36, *P* < 0.01) domains (Fig. 4C). Interestingly, if we group the results by the type of radiotherapy, we noticed that the patients who underwent IMRT had a decrease of 6.75 points in the function domain, while the patients who underwent custom blocking only had a decrease of 1.17. This represents a difference of 5.58 points (*t*-test, *P* = 0.03).

## Significance of results

Physicians select a treatment that offers the best survival outcome with the least treatment-related toxicities [15] while considering age and underlying comorbidities to determine the intensity of therapy [16]. Therefore, it is of increasing interest to also consider the patients’ QoL after receiving treatment. For this reason, the use of QoL questionnaires has become a useful tool to evaluate the subjective side effects of the treatment on the patients’ day-to-day activities.

Factual scores of post-treatment QoL questionnaires may be deceiving as they do not follow the lead of each individual [17]. Therefore, measuring the differences between pre- and post-treatment questionnaire scores may reveal the true benefit/detriment of the intervention on their QoL. Moreover, there is evidence that composite QoL scores may not be accurate as they mask potential changes in domain-specific scores [18]. Therefore, the analysis of each individual domain may be a more sensitive tool to evaluate subtle changes in QoL. With this approach, we were able to see that patients reported lower scores in the UW-QoL questionnaire post-treatment when asked about their perception of appearance, taste, and saliva. Similar to our findings, several other studies have reported comparable detrimental effects in these domains in patients who underwent treatment with high-dose cisplatin chemotherapy and/or radiotherapy [2, 19, 20]. Moreover, as our team previously reported [6], the most common side effects observed in our patients while taking erlotinib for 7 weeks were grade 1–2 skin rash, diarrhea, and nausea presenting in 63%, 47%, and 31% of the patients, respectively. Interestingly, in a phase II study of erlotinib for recurrent or metastatic HNC squamous cell carcinoma, the authors reported similar findings with rash and diarrhea presenting in 79% and 37% of the patients while taking erlotinib for 8–48 weeks [21]. Due to the similar findings of both studies, in spite of the differences in drug consumption time frames, we question if side effects observed in the long-term of our patients were secondary to the addition of erlotinib vs chemotherapy alone. A phase III study reported that an average of 41% of patients undergoing chemotherapy presented a grade 3–4 toxic effect (neutropenia and thrombocytopenia) vs 0% with erlotinib [10], while another phase II study concluded that the combination of erlotinib with chemoradiation does not reduce the patient’s QoL compared to chemoradiation alone [22]. Additionally, Wijers et al. noted a long-term xerostomia that occurred in 64% of the patients post radiation [23], while Dijkema et al. reported a rate of 56% [24] and Rades et al. a rate as high as 73% [25]. Similarly, the rate of xerostomia in our own cohort was over 68% throughout the 5-year follow-up. Likewise, in the literature, long-term dysphagia has been noted at a rate of 3% to 21% [26], while our patients’ cohort presented a 30% rate of dysphagia by year 5. Moreover, Lambert et al. reported that 23% of the patients treated with chemoradiation developed late toxicities, including percutaneous endoscopic gastrostomy (7%), persistent dysphagia (6%), pharyngoesophageal stenosis (2%), and permanent tracheostomy (8.5%) [27]. Another caveat to our toxicity findings includes the fact that we used a different delivery method of cisplatin, namely, intra-arterial, with peripheral neutralization. In a study conducted by Ono et al., 3.8% of the patients presented with late toxicities (chondronecrosis and severe dysphagia) within, but not after, 5years following intra-arterial cisplatin with concurrent radiation treatment [28]. Although we did not observe significantly higher side effects than other studies employing chemoradiation to treat locally advanced head and neck cancers, it is possible that the use of an intra-arterial method of delivery of cisplatin enabled patients to tolerate a biologic therapy better and kept long-term side effects to historical levels.

Although different modalities have been implemented to spare normal structures and to reduce the negative impact of radiation in patients with HNC (i.e., compensators, IMRT, IGRT), dysphagia still remains a major side effect [29, 30]. Interestingly, with newer techniques such as IMRT, while limiting swallowing impairment, patients still present with dysphagia when compared with their pre-treatment function [30]. One of the most common side effects observed in our patients was xerostomia, regardless of the type of radiotherapy received. These patients, as with other studies, required fluids while eating or complained about having food suck in their mouth, as well as having the perception of “dry mouth.” However, this may not be related to the actual salivary flow. In a study conducted by Frazén et al., the authors observed that the subjective discomfort during swallowing was not correlated with an objective salivary flow [31]. In parallel with this idea, our patients had lower scores in the functional and composite domains in the dysphagia inventory post-treatment compared to their baseline.

Considering all the side effects that come when receiving multiple treatments, it would be natural to ask if it is worth the risk of having functional detriments while opting for organ-preserving treatments. To try to answer this question, there is increasing evidence that the addition of different organ-preserving treatments improves overall survival compared to a single agent [4–6, 32]. Additionally, in our study, fewer patients had complaints about pain, speech, and anxiety at the time of answering the UW-QoL questionnaire post-treatment. This suggests that patients can cope with the functional limitations that may come with organ preservation. Following this idea, we can see that the post-treatment FACT-H&N questionnaire scores from our patients, although not statistically significant, display a tendency of improvement in the physical and emotional domains while presenting a decrease in the functional domain. Furthermore, there is evidence that FACT-H&N scores are more sensitive to gains than to losses [33]. Therefore, only minimal increases in scores are sufficient for the patients to sense any true benefit from their treatment, especially when their swallowing ability is affected [34]. As noted by Ojo et al., most QoL assessment instruments are designed to cover “site-specific” and “symptom-specific” issues instead of “treatment-specific” issues [35]. However, the use of a single instrument, although designed to be “stand-alone,” may not cover every domain. For this reason, the choice of QoL instruments should be based on the domains of interest and, ideally, overlap several instruments to have a robust evaluation.

As our data, as well as other studies, suggests that the combination of erlotinib with chemoradiation is a viable option to treat HNC malignancies [32, 36], we decided to compare the overall survival of our patients with other treatment regimens. With our data, the 1-, 3-, and 5-year overall survival rates of our patients, regardless of comorbidities, were 83%, 67%, and 55%, respectively. To put this in perspective, Jin-Hau et al. reported a 5-year overall survival of 50.37% when using an aggressive treatment of surgery, radiotherapy, and/or chemotherapy in patients with a low (< 10) Charlson comorbidity index (CCI) score and 24.53% with high (≥ 10) CCI scores [37]. Similarly, Chang et al. compared the 5-year overall survival of patients with advanced stages of HNC cancer treated who underwent re-RT alone (13.34%), CT alone (21.13%), CCRT (20.09%), and surgery ± RT/CT (37.93%) [38]. However, Le et al. did not find a difference in overall survival benefit in adding erlotinib to platinum–docetaxel chemotherapy vs placebo in patients amenable for surgical resection [39]. Therefore, as newer studies have suggested modification or de-escalation of treatment regimens to help reduce their impact on the patients’ QoL [7], it is of particular importance to have proper patient selection as some trials have failed to improve outcomes while trying to produce less toxicity [15]. For instance, patients that are HPV-positive have been shown to have a better response to chemoradiotherapy than patients that are HPV-negative [40–42]. This has raised the possibility of de-escalating the treatment regimen for these patients [41]. However, several trials have failed to demonstrate better outcomes with a de-intensified regimen [43, 44]. Perhaps, an improved understanding of predictive biomarkers will enhance how we select patients for organ-preserving treatments [45].

One limitation of our study is the number of patients included for analysis and the frequency with which we implemented the QoL questionnaires. As mentioned in the “Methods” section, we reviewed the QoL of patients who previously enrolled in an open-label, non-randomized, phase II study by Dr Rao. Hence, the study team limited the enrollment to the minimum necessary for feasibility of answering the primary objective of the study. Nonetheless, although our data may be limited in terms of generating firm conclusions, our results display a tendency similar to comparable treatment regimens noted in the literature.

### Supplementary Information

Below is the link to the electronic supplementary material.

Supplementary file1 (JPG 830 KB)

Supplementary file2 (JPG 656 KB)

Supplementary file3 (JPG 624 KB)

## Data Availability

All data generated or analyzed during this study are included in this published article and its Supplementary information files.
