# Supplementary figures and images for: Quality of life following treatment with intra-arterial cisplatin with concurrent radiation and erlotinib for locally advanced head and neck cancer
Source: Support Care Cancer. 2024 Jan 9;32(2):93. doi: 10.1007/s00520-023-08286-1 (PMC10776718; doi:10.1007/s00520-023-08286-1)

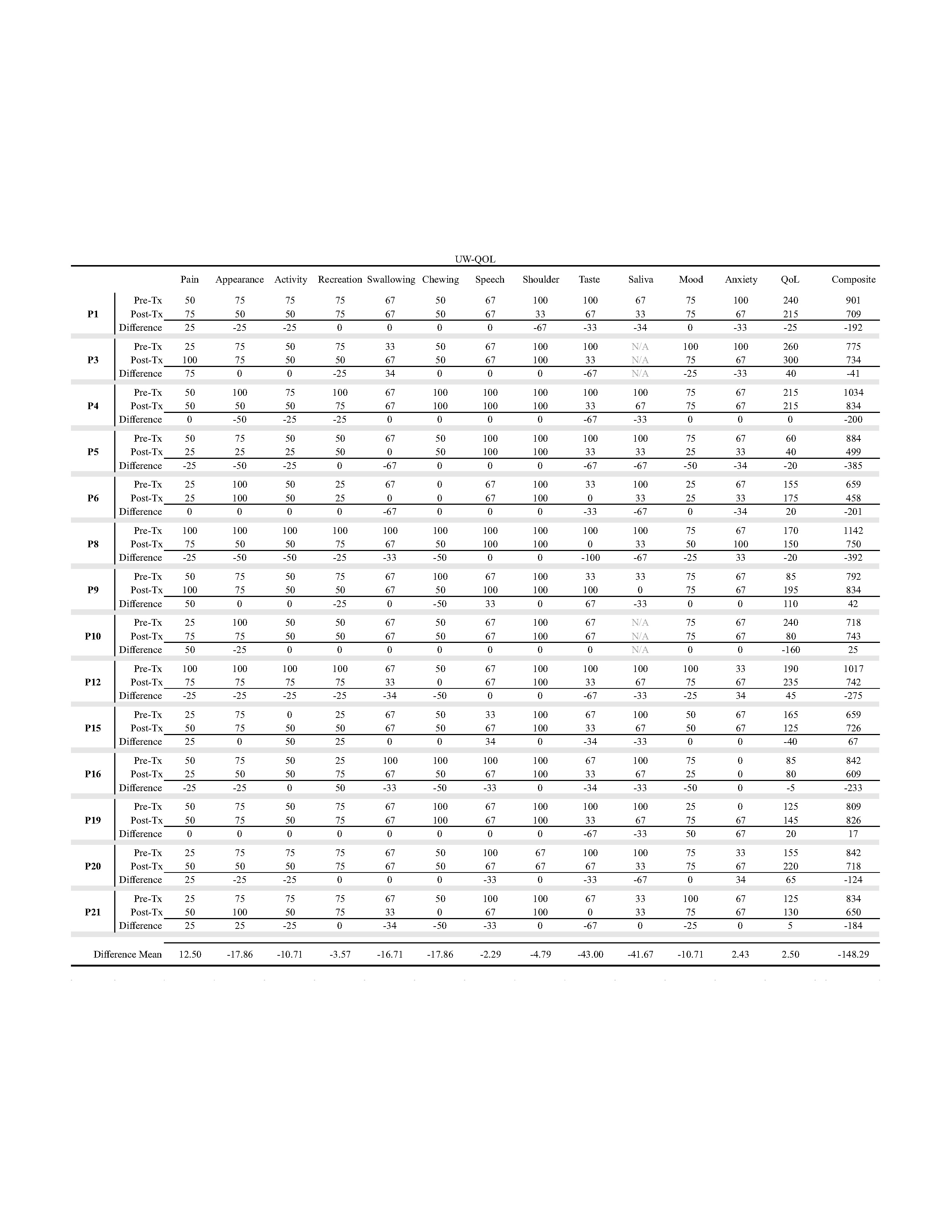

Supplement: Supplementary file 1 — Supplementary file1 (JPG 830 KB) [file 520_2023_8286_MOESM1_ESM.jpg]

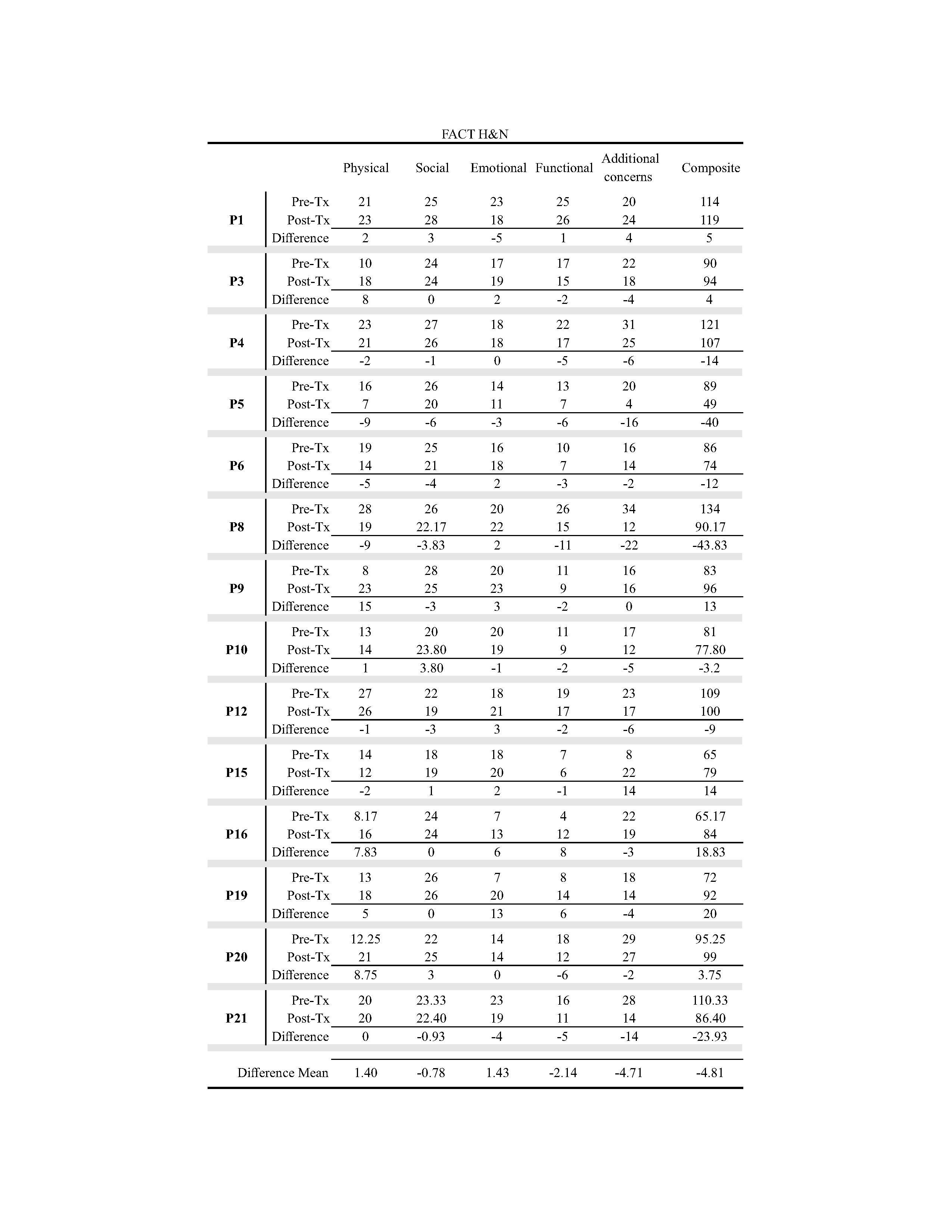

Supplement: Supplementary file 2 — Supplementary file2 (JPG 656 KB) [file 520_2023_8286_MOESM2_ESM.jpg]

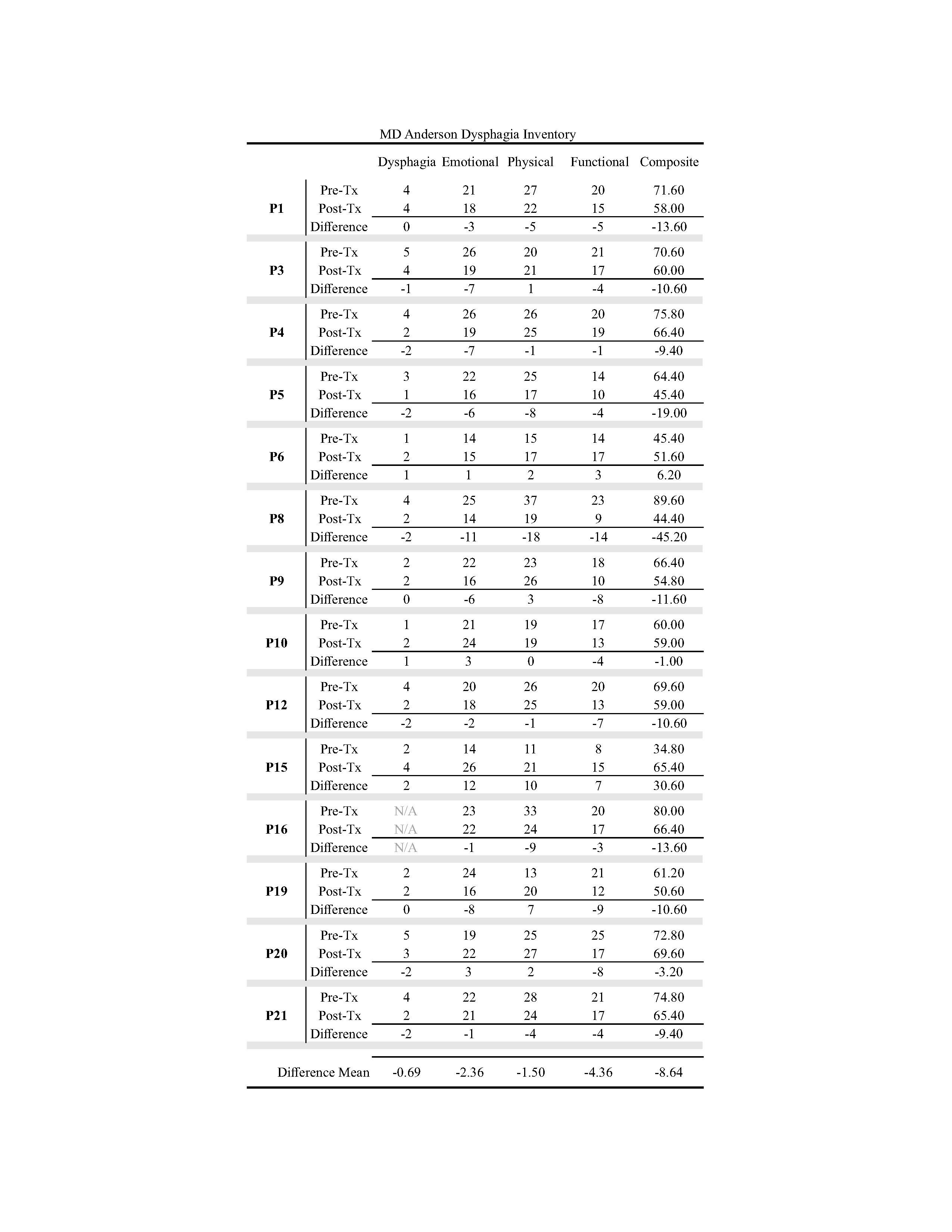

Supplement: Supplementary file 3 — Supplementary file3 (JPG 624 KB) [file 520_2023_8286_MOESM3_ESM.jpg]
